# The Synergistic Effect of Triazine and Phosphaphenanthrene Units on the Physico-Chemical Behavior of Polyimides

**DOI:** 10.3390/molecules28104072

**Published:** 2023-05-13

**Authors:** Irina Butnaru, Mariana-Dana Damaceanu

**Affiliations:** Electroactive Polymers and Plasmochemistry Laboratory, “Petru Poni” Institute of Macromolecular Chemistry, Aleea Gr. Ghica Voda 41A, 700487 Iasi, Romania; butnaru.irina@icmpp.ro

**Keywords:** polyimide, 1,3,5-triazine, DOPO, “black” appearance, fluorescence, electrochemistry

## Abstract

With the aim to develop polymers with appealing, multifunctional characteristics, a series of polyimides were designed by anchoring 9,10-dihydro-9-oxa-10-phosphaphenanthrene 10-oxide (DOPO) units on the main polymer chains containing 1,3,5-triazine and several flexible moieties, such as ether, hexafluoroisopropylidene, or isopropylidene. A detailed study was conducted to establish structure–property correlations, with a focus on the synergistic effectiveness of triazine and DOPO moieties on the overall features of polyimides. The results evidenced good solubility of the polymers in organic solvents, their amorphous nature with short-range regular-packed polymer chains, and high thermal stability with no glass transition temperature below 300 °C. Spectrophotometric measurements revealed the existence of a strong charge transfer complex in these polymers that led to a “black” appearance, which generated broad absorption bands spanning on the overall visible range. Nevertheless, these polymers displayed green light emission associated with 1,3,5-triazine emitter. The electrochemical characteristics of the polyimides in solid state demonstrated their strong *n*-type doping character induced by three different structural elements with electron-acceptance capability. The useful properties of these polyimides, including optical, thermal, electrochemical, aesthetics, and opaqueness, endow them with several possible applications in the microelectronic field, such as protecting layers for the inner circuits against UV light deterioration.

## 1. Introduction

Polyimides belong to the well-known class of high thermostable polymers which have continuously attracted the attention of academia and industry due to their unique and appealing combination of physical and chemical properties. Since their discovery in 1908, polyimides have been subjected to various modifications in order to render the designed polymeric architectures with targeted properties for a wide range of applications. Relevant reviews that have described the synthetic pathways for the development of polyimides, their physico-chemical characterization, as well as the diverse range of applications have been published over the last 10 years [1,2,3,4,5,6,7]. The characteristics of classical all-aromatic polyimides include high-temperature resistance, mechanical and chemical stability, elevated glass transition temperature, variable optical transmittance, as well as low dielectric constants. All these properties can be tailored by altering the architecture of the polymer chains, and they strongly depend on the functional groups inserted in the macromolecular backbones which also influence the molecular weight, solubility, crystallinity, intermolecular forces, or electron conducting/storage ability in addition to the above-mentioned properties. All these characteristics make the newly developed polyimides valuable candidates for a wide range of applications in different forms, such as film, fiber, nanofiber, membrane, foam, adhesive, or coating in aerospace, electronics, automotives, medicine, information recording and new imaging technology, modern paper, solar cells, or other green energy fields [8]. Thus, researchers have customized the polyimide macromolecular chain in order to obtain materials with specific properties for targeted applications. The approaches that have been undertaken have mainly consisted of incorporating various moieties into the starting monomer’s structure (kink, spiro, cardo, cycloaliphatic, bulky, fluorinated, hetero, carbazole, perylene, chiral, nonlinear optical, or unsymmetrical units), copolymerization reactions, functionalization of the already obtained polyimides, or use of different fillers towards nanocomposite-based polyimide material development [5,7,9,10,11,12,13,14]. It has been proven that the incorporation of aromatic ether and isopropylidene units enables increased processability and lowered glass transition temperature of the corresponding polyimides, while maintaining increased thermal stability [15,16,17,18]. On the other hand, bulky hexafluoroisopropylidene moieties are known for their ability to enhance flame resistance properties and gas separation characteristics through increased free volume, as well as to improve the electrical insulation ability [16,19]. 

Although not often incorporated into polyimides, 1,3,5-triziane can also lead to high-performance materials with special properties; 1,3,5-triazine, as part of nitrogen-containing heterocycles, is used for the synthesis of bioactive compounds, and has been successfully employed as cross-linking agents for bioconjugation, dehydrating agents, building blocks for star-shaped structures/hyperbranched polymers, as well as starting materials for various s-triazine derivatives [20]. In addition, it has been involved in the development of herbicidal, fungicidal, and insecticidal products and possesses antitumor activities. Due to its nitrogen-rich structure, 1,3,5-triazine has gained the interest of researchers for obtaining energetic materials and explosives, or efficient growth of nitrogen-doped graphene on a large scale [21]. As a consequence of their good electron-acceptor ability, triazine derivatives have been widely used as electron transport layers in organic light emitting diodes, thermally activated delayed fluorescence emitters, and donors in bulk heterojunction solar cells [22,23,24,25]. When triazine derivatives have been incorporated into polyimide structures, new interesting materials have been obtained for use as sodium-ion battery anodes [26], highly efficient removal of Ni(II) from aqueous solution [27], mixed-matrix membranes for CO_2_/CH_4_ separation [28], covalent organic frameworks for high-performance Li storage [29], or as cathodes for sodium storage [30]. 

One approach to supply industrial needs for high-temperature and flame-retardant materials consists of the development of phosphorus-based polyimides. It has been proven that the incorporation of phosphorus into polymer chains (such as phosphine oxide or phosphonate) led to improved solubility in organic solvents, flame retardancy, atomic oxygen resistance, and adhesion to metals, while maintaining their desirable characteristics. The thermal stability endowed by the incorporation of phosphinate linkage [–O–(O=)P–O–C] into a cyclic form is higher than that of a phosphonate type [–O–(O=)P–O–] based polymer [31]. Thus, recently, several phosphorus-containing polyimides have been reported with improved physico-chemical properties through monomer functionalization with flexible units [16,32,33,34] or by using copolymerization techniques [35,36]. Particular interest has been devoted to the development of phosphorus-based polyimides for use as proton exchange membranes [37,38], or polyimide composites with pristine or functionalized polyhedral oligomeric silsesquioxane for atomic oxygen resistance applications [39,40,41].

Polymers containing 9,10-dihydro-9-oxa-10-phosphaphenanthrene 10-oxide (DOPO) have attracted interest due to the DOPO bulky structure which enables free conformational stress and prevents polymer chains packing [42,43]. When inserted into polyimide chains, DOPO moiety can improve polymer solubility, thermal stability, and flame retardancy [44]. From our knowledge, the number of studies in the literature reporting on DOPO-containing polyimides is relatively low [31,45], and they rely mainly on describing the influence of DOPO derivatives on solubility, crystallinity, thermal, and flame retardant properties. Lately, the studies on DOPO-containing pristine polyimides have focused on analyzing the gas-transport properties, optical or flame-retardant properties [43,46].

Despite the numerous articles based on the synthesis of polyimides published so far, none of them have focused on the incorporation of triazine and DOPO heterocyclic units in the same macromolecular chain. Therefore, this study attempts to highlight the synergistic effect of DOPO and triazine on the physico-chemical properties of a series of polyimides which also incorporate several flexible linkages. To this aim, a diamine monomer containing DOPO-substituted triazine was used in polycondensation reaction with several dianhydrides which incorporate bridges of variable flexibility. We explored, in detail, the influence of this bulky unit on the overall polyimide properties, including thin films morphology, photo-optical characteristics, thermal stability, and electrochemical features.

## 2. Results and Discussions

**Scheme 1:**
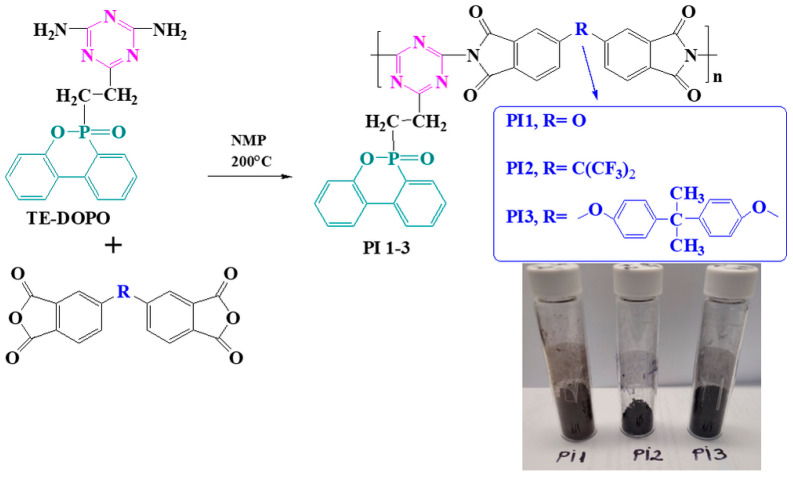
Synthesis of DOPO-substituted triazine-containing polyimides PI1–PI3 (the inset shows the images of the obtained polyimide powders).

With the aim to develop polymers with useful characteristics for nowadays technologies, the classical polyimide structure was modified by anchoring DOPO on the 1,3,5- triazine unit of the main polymer chains. To enhance the solubility and polymer processability by wet methods, additional moieties with various degrees of flexibility were incorporated into the polymer structure, taking care to maintain the high thermal stability and other inherent properties of classical polyimides. To this aim, the diamine TE-DOPO containing DOPO-substituted triazine was used in the polycondensation reaction with three commercial aromatic dianhydrides to synthesize the desired polyimides, PI1–PI3, according to Scheme 1. Due to the diamine insolubility in NMP at room temperature, the polycondensation reaction was conducted in a single step, without the isolation of the intermediate polyamidic acid.

### 2.1. Structural Characterization

**Figure 1:**
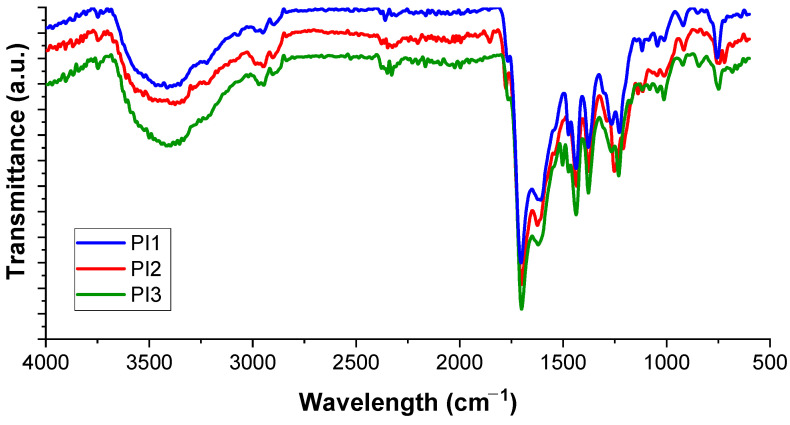
Stacked FTIR spectra of triazine– and DOPO–containing polyimides PI1–PI3.

The structural identification of the obtained polyimide powders was accomplished by FTIR and NMR spectroscopies. The FTIR spectra revealed the existence of the characteristic absorption bands of the newly formed imide cycle at: 1777–1764 cm^−1^ (C=O asymmetric stretching vibration), 1704–1700 cm^−1^ (C=O symmetric stretching vibration), 1378–1377 cm^−1^ (C–N stretching vibration), and 754–717 cm^−1^ (imide ring deformation). The absorption bands identified at about 1542–1540 cm^−1^ were attributed to the heterocyclic triazine unit. Evidence supporting the maintenance of the cyclic DOPO groups in the polyimides was provided through the appearance of some characteristic absorption bands, as follows: P–Ar at about 1475–1472 cm^−1^, P–CH_2_ at 1436–1434 cm^−1^, P=O at 1146–1140 cm^−1^, and P–O–Ph at 920–916 cm^−1^. The absorption bands due to C–H aliphatic bonds from the ethylidene bridge which connects the two heterocycles were found in the ranges of 2965–2951 cm^−1^ and 2894–2898 cm^−1^, providing further evidence for the existence of the unaltered bulky DOPO-substituted triazine unit in the macromolecular chains of the polyimides. Aromatic C–H and C–C linkages of the aromatic rings were identified at approximatively 3080–3060 cm^−1^ and 1608–1606 cm^−1^, respectively. The specific flexible group from the diimide segment of each polymer was identified at 1227 cm^−1^ due to the absorption of aromatic ether units in PI1, 1209 cm^−1^ due to the absorption of CF_3_ moieties in PI2, and 2965 cm^−1^ and 2898 cm^−1^ due to the absorption of aliphatic C-H linkage belonging to the C(CH_3_)_2_ group of PI3 (Figure 1).

An important aspect provided by the FTIR spectra relates to the absorption bands which appeared in the ranges of 3409–3401 cm^−1^ and 1637–1623 cm^−1^ which can be attributed to carboxylic and amide groups absorptions, respectively. These suggest the occurrence of an incomplete imidization of the intermediary polyamidic acids. We presumed that when polyamidic acids were formed, certain intermolecular interactions occurred between the carboxylic acid units from the main chains and DOPO pendant unit (through –OH and P=O), which impeded the formation of a fully-cyclized polyimide, despite the long reaction times and catalyst used in the polycondensation reactions. The incomplete cyclization of the intermediary polyamidic acids to the corresponding fully cyclized polyimide forms is a known phenomenon encountered for polyimides bearing bulky pendant groups where a 100% degree of imidization is rarely found [47,48]. 

**Figure 2:**
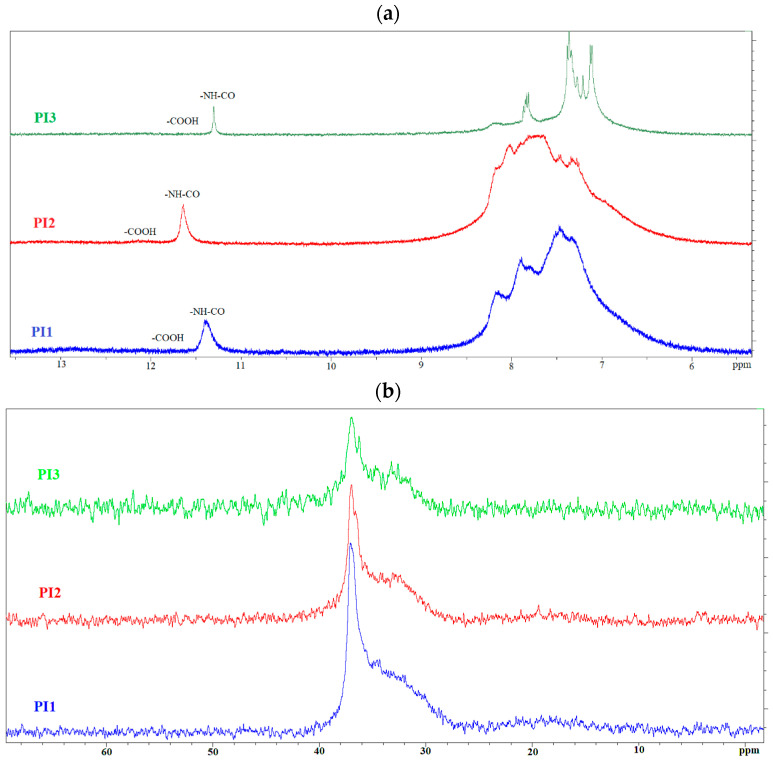
^1^H-NMR (**a**) and ^31^P-NMR (**b**) spectra of triazine– and DOPO–containing polyimides PI1–PI3.

The NMR spectra provided additional support for the FTIR data discussed above. In the case of the polyimides PI1 and PI2, which display a more rigid structure of the diimide segment, the aromatic protons coming from the diamine and diimide segments appear as broad overlapped signals, in the ranges of 7.31–8.16 ppm (PI1) and 7.25–8.16 ppm (PI2). Polyimide PI3, which contains a longer flexible unit, exhibits a slightly clearer spectrum with peaks separated in two regions: 8.3–7.18 ppm and 7.36–7.12 ppm (Figure 2a). The different pattern of the ^1^H-NMR spectrum of this polymer may be associated with the higher flexibility induced by the presence of 6H and ether units, which reduce the chain packing to a lower degree compared to PI1 and PI2, where intermolecular interactions are predominant. The ^1^H-NMR spectra of the polyimides were compared to that of the starting diamine monomer and the results indicated its full incorporation into the corresponding polymers due to the disappearance of characteristic amine protons from 6.55 ppm. Still, in line with FTIR findings, ^1^H-NMR spectra also revealed the existence of residual polyamidic acid groups in all polymers due to the broad peak found at about 13.15–12.10 ppm and the sharp peak centered at 11.64–11.31 ppm, which were attributed to the carboxylic and amide protons, respectively.

The ^31^P-NMR spectra also confirmed the correct structure of the synthesized polyimides through the presence of a main sharp peak at about 36.98–37.04 ppm associated with the phosphorus of the polyimide chains, and a broad, weak shoulder-like peak in the range of 32–33.08 ppm, which was tentatively attributed to the non-cyclized polyamidic acid segments (Figure 2b). The broadness of the latter provides evidence for the involvement of the P=O unit in intermolecular interactions.

Due to the incorporation of the bulky pendant DOPO segment in the side chains and of flexible ether, hexafluoroisopropylidene, or isopropylidene units in the main chains, improved organo-solubility and processability of the synthesized polyimides is expected. To verify this, solubility tests were performed using 5 mg of polyimides in 1 mL solvent. The results evidenced a very good polymer solubility in polar organic solvents (NMP, DMAc, DMF, and DMSO). In the case of less polar solvents, such as THF, chloroform, acetone, or dichloromethane, PI1–PI3 were insoluble even on heating (Table 1). The good solubility at room temperature in organic polar solvents is attributed, on one hand, to the polymers non-coplanar structure induced by the presence of the voluminous DOPO-substituted ethylidene unit from the side chain and, on the other hand, to the flexible groups from the diimide segment. The synergetic effect of these structural modifications led to polymer conformations which enabled the penetration of small solvent molecules through the chains and polymer solubilization. 

Owing to the good solubility in polar organic solvents, evaluation of the average molecular weights of polyimides was possible by using GPC measurements. The data revealed relative high values for the investigated parameters: Mn had values between 151,700 and 301,700 g/mol, Mw was in the range of 354,500–693,900 g/mol, while the polydispersity index was between 2.19 and 2.34 (Table 2). 

Apart from this, GPC diagrams evidenced signals for the residual polyamidic acid segments with low Mn values, in the range of 6500–7700 g/mol (Mw of about 7100–8200 g/mol and polydispersity index between 1.07 and 1.1). Thus, contents of about 12, 10, and 8 repeating units of polyamidic acid segments relative to approx. 565, 912, and 716 imide segments were found, which means a conversion of 97.9%, 98.9%. and 98.9% of the polyamidic acid to the imide structure for PI1, PI2. and PI3, respectively.

### 2.2. XRD and SEM Studies

**Figure 3:**
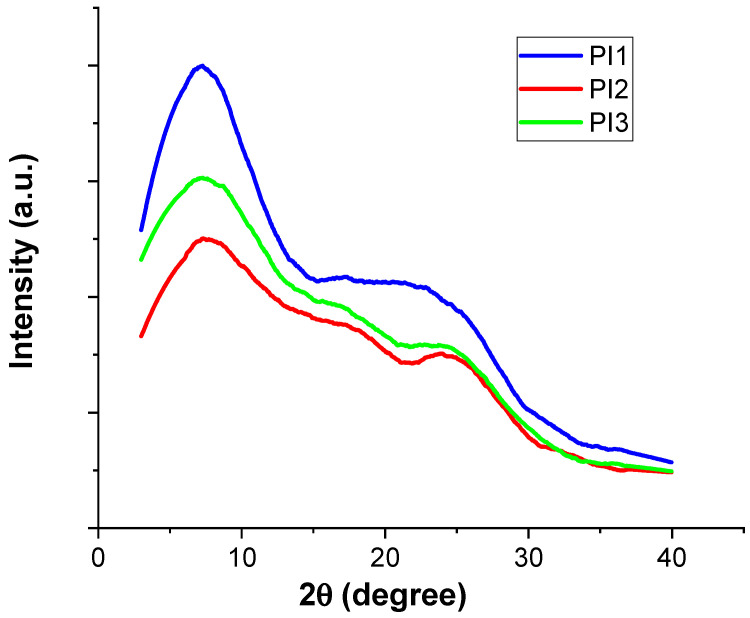
WAXD patterns of the polyimides PI1–PI3.

Due to the black appearance of the polyimide powders (as can be seen in Scheme 1), XRD measurements were registered to analyze the possible crystalline nature of the polymers. The broad X-ray diffractogram of the polyimides (Figure 3) in the range of 15–25° indicate their amorphous nature. The two overlapped halos identified in this region may be associated with the mixture of the two polymer structures, i.e., polyimide and polyamidic acids. The peak found at smaller diffraction angles in all polyimides (approx. 7°) is tentatively attributed to the changes in local order (regular repeating distances), i.e., short-range regular packing of polymer chains with no crystalline features [49]. The amorphous nature of these polyimides is due to the presence of the bulky unsymmetrical DOPO-substituted triazine moiety in the diamine segment and of flexible units (ether, hexafluoroisopropylidene, or isopropylidene) in the diimide segment. All of these structural units disturb the overall regularity and hinder the arrangement in crystalline domains of polyimide chains [50].

**Figure 4:**
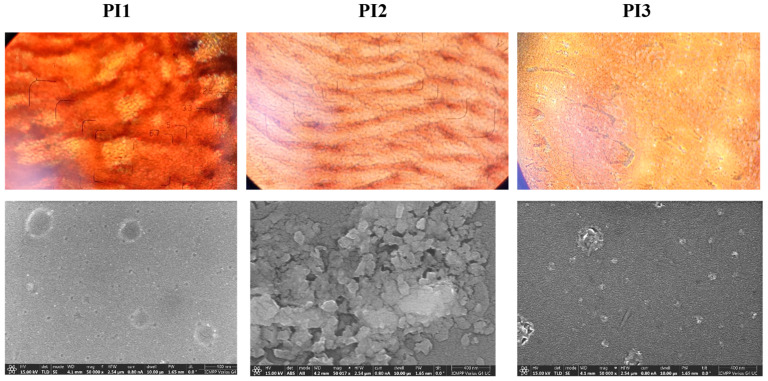
OM ((**top**), magnification 20×) and SEM (**bottom**) images of PI1–PI3 film surfaces.

Starting from the XRD results, it was anticipated that some solid-state organization may occur in thin films obtained from these polymers. Thus, coatings of PI1–PI3 were obtained on glass plates from polymer solutions in NMP by using the drop-casting technique, after gradual annealing until 250 °C. To further evaluate the films’ morphology, optical microscopy (OM) visualizations were first involved. The photographs taken at a magnification of 20× evidenced the formation of continuous and homogeneous films, with similar morphologies for all polymers (Figure 4, top). Although the polyimides’ structures contain different diimide segments, no obvious influence of this structural parameter on the morphological features of the films was noticed.

The capability of the polymer chains to arrange into more defined assemblies was observed by performing scanning electron microscopy (SEM) investigations. Thus, when the surfaces of thin films were analyzed, similar morphological features were found for all polymers, as in the case of OM. The rigid rods of the polymer macromolecules generated by the imide-triazine framework mostly experienced π–π stacking interactions during film preparation under heating, leading to a grain-like morphology (Figure 4, bottom). However, from a certain level, the primary morphological entities arranged in bigger assemblies, as well-observed in the case of PI2. Most likely, surface tension constraints occurred that forced the grains to organize into clusters having a sheet-like shape and variable dimensions. It should be noted that this type of solid-state organization has not been previously reported for DOPO-containing polyimides, which prompted us to consider that a strong contribution to it originates from triazine that through the synergetic effect with imide and DOPO induced a close macromolecular packing.

### 2.3. Thermal Properties

**Figure 5:**
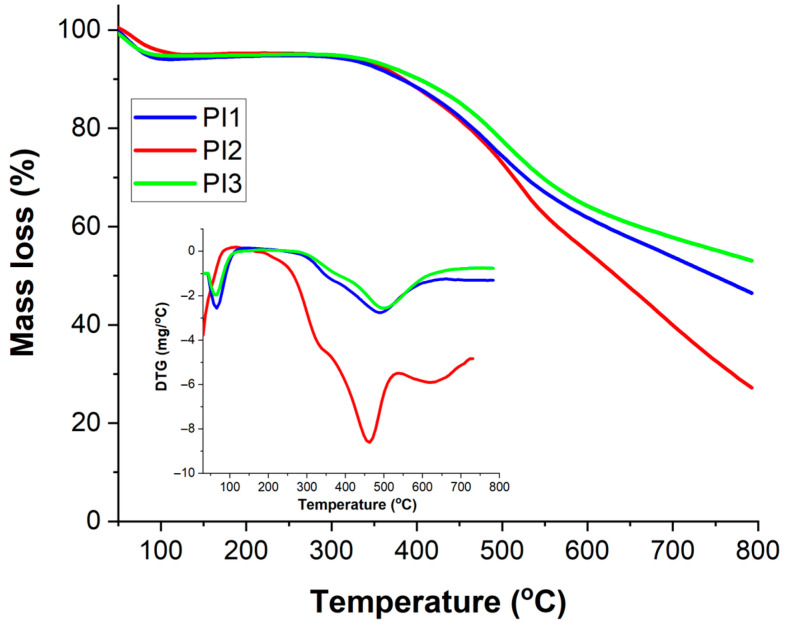
TG and DTG curves of polyimides containing DOPO-substituted triazine PI1–PI3.

Differential scanning calorimetry and thermogravimetric investigations were used to analyze the thermal stability of the polymers, and the obtained data are shown in Table 2 and Figure 5. The glass transition temperature for all polymer powders could not be detected in the temperature limit of the experimental setup, so that no Tg was registered until 300 °C for any of the polyimides. Most likely, this phenomenon is present at a higher temperature due to the high degree of aromaticity of the main polymer chains and the presence of intermolecular interactions between macromolecules in solid state.

The initial decomposition temperature of polyimides, taken as 5% weight loss (T_5_) registered values in the range of 336–352 °C, in close correlation with the nature of the flexible unit from the dianhydride fragment. The highest value of T_5_ was obtained for PI2 which incorporates 6F units, while the lowest one was registered for PI1 containing ether bridges in the main chain. However, the latter displayed an initial decomposition temperature close to that found for PI3 with isopropylidene moieties in the diiimide segment (339 °C). These values are comparable to those obtained for related DOPO-containing polyimides with the same flexible linkages [43], and are slightly lower than those reported for fully aromatic conjugated triazine-based polyimides [30]. The degradation mechanism followed the same trend for polyimides PI1 and PI3, employing one major decomposition step, while PI2, incorporating hexafluoroisopropylidene units in the polymer structure, exhibited an additional decomposition step, which, in this case, was attributed to the scission of the fluorinated moieties. A shoulder-like peak can be observed in the DTG curves at about 369, 338, and 376 °C for PI1, PI2, and PI3, respectively, which can be assigned to the decomposition of the voluminous triazine-ethylidene-DOPO fragment [42]. The main step of decomposition in all polymers was found in the range of 490–516 °C, and was correlated with the degradation of the main macromolecular chains. The char residue values at 800 °C were higher in the case of PI1 and PI3 (W_800_ = 46.44% and 53.05%, respectively), and they were comparable to the values recorded for related DOPO-containing polyimides [43,45]. As expected, the introduction of DOPO-substituted triazine unit along with flexible linkages in these polyimides preserved an increased thermal stability, and therefore, the present polymers are suitable for applications that require resistance to high temperatures. 

### 2.4. Photo-Optical Characteristics

**Figure 6:**
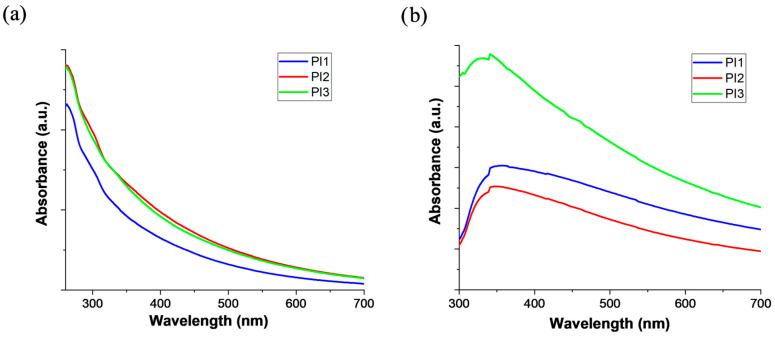
UV-Vis absorption spectra of PI1–PI3 in DMSO solutions (**a**) and in solid state (**b**).

Spectral characterization by UV-Vis absorption and fluorescence was carried out with the aim to evaluate the electronic transitions induced by the triazine and imide heterocycles in polymers PI1–PI3, and also the presence of molecular interactions. Figure 6 illustrates the electronic absorption spectra of these polyimides recorded in DMSO solution and in solid state.

According to Figure 6, the absorption bands of the studied polyimides are weak, in the form of shoulders, and difficult to localize, with maxima at wavelengths of about 270, 290, and 300 nm. These overlapped high-energy absorptions can be attributed to the π–π* and *n*–π* transitions in the imide, triazine, DOPO, and benzene rings, according to previous reports [51,52]. Although the structural variation in the diimide segment tailored the macromolecular conformation and planarity of PI1–PI3, this had little effect on the electronic transitions in these polymers since no obvious shifts in the absorption maxima were observed. It should be emphasized that all polyimides showed in their UV-Vis absorption spectra a tail-like band spanning the overall visible domain, which may suggest the existence of the charge transfer complex (CTC) in these polymers.

As thin films, the present polyimides showed different photo responses compared to the isolated molecules in liquid phase, with broader absorption bands spanning the overall visible absorption range, which apparently displayed maxima shifted to higher wavelengths (Figure 6b). Thus, the absorption maximum was noticed at 352, 347, and 329 nm for PI1, PI2, and PI3, respectively. These data indicate that the optical changes in solid state are considerably affected by the chemical structure of the diimide segment. Accordingly, PI1 displays the highest co-planar conformation and packed polymer chains due to the more rigid shape of the ODPA-derived segment, followed by PI2 with its bulky hexafluoroisopropylidene unit that reduced these features to some extent. Conversely, PI3 is characterized by the most non-planar arrangement and the lowest solid-state packing in the series, clearly reflected in the most hypsochromic shifted UV-Vis absorption band registered for this polymer. 

**Figure 7:**
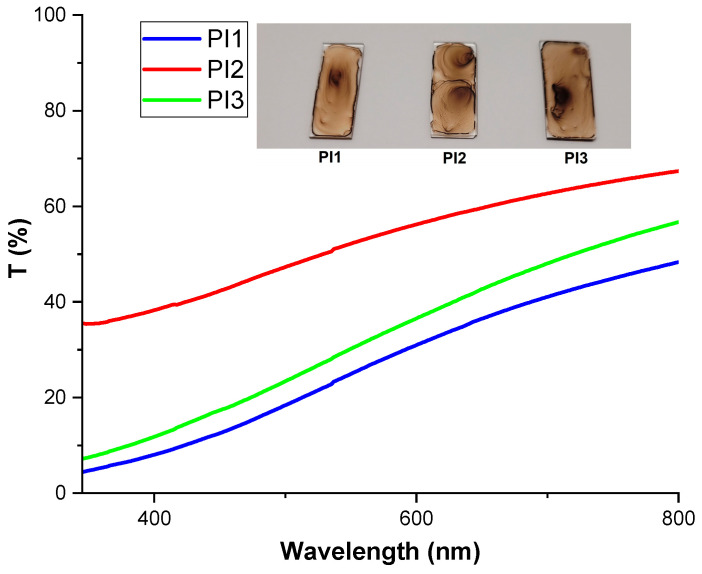
Transmittance spectra of PI1–PI3 films.

Another important aspect to be mentioned here is related to the obvious intensification of the CTC band for these polymers, mostly induced by the better planarity of the macromolecules in solid state, which definitely favors the CTC transitions. Usually, the colors of polyimide films are much deeper when CTC formation is stronger. As mentioned earlier, our polymer films are brown-black in appearance and this correlates well with our assumption on CTC strength. Such a characteristic of “black polyimides” was previously observed for other polyimides derived from nitrogen-containing diamines [53]. According to the transmittance spectra shown in Figure 7, these PI films, especially PI1 and PI3, experience a significant decrease in the optical transmittance on overall UV-Vis spectral range. For instance, the thin film of PI1 is almost opaque at the wavelength of 500 nm, showing 18% transmittance at 500 nm and 31% at 600 nm. The highest transmittance of PI2 can be explained by the presence of low polarizability trifluoromethyl groups that reduces the CTC formation through steric hindrance and inductive effect leading to weakening chain-to-chain cohesive forces.

These appealing optical properties endow the present polyimides with a large applicative prospect in fields where high heat resistance, chemical inertness, aesthetics, and opaqueness are all together required, for example, to cover flexible printing circuit boards in order to protect the inner circuits from UV light deterioration. Although the present polymers cannot be processed in free-standing films, further modification via blending technique can be promising to suppress this drawback and benefit of their valuable properties. The information on this subject will be reported in future work.

**Figure 8:**
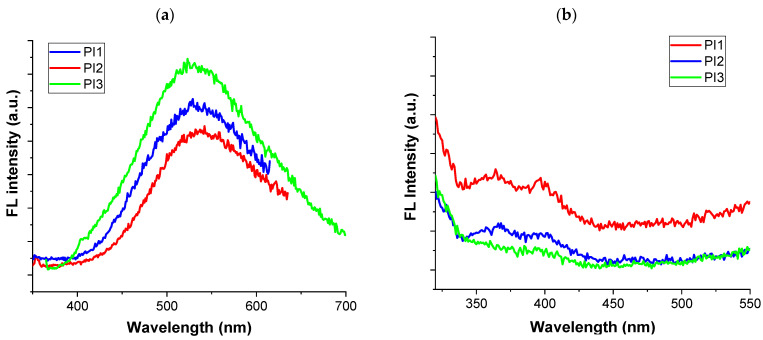
Fluorescence spectra of PI1–PI3 in DMSO solutions (**a**) and in solid state (**b**).

Since 1,3,5-triazines as electron acceptor units form the most attractive building blocks in the development of thermally activated delayed fluorescence (TADF) materials with high efficiencies and long operating lifetimes in OLEDs [54], it is of particular interest to explore the fluorescence properties of PI1–PI3 having this heterocycle as a constitutive structural element. To the best of our knowledge, the synergistic effect of 1,3,5-triazine and DOPO units on the fluorescence properties of polyimides is still unknown. Thus, the fluorescence behavior of the present polymers in DMSO solutions and condensed state (thin films) were investigated. A well resolved fluorescence band was recorded in the spectral pattern of all polyimides, in the range of 528–539 nm, which indicated their ability to release green light (Figure 8a). This emission was associated with 1,3,5-triazine since this heterocycle is a well-known electron-deficient moiety used in green and blue TADF emitters [54]. Previous studies on related polyimides containing DOPO units have highlighted that their emission in polar solvents occurs in the UV-blue spectral range [46], which definitely provides support for our statement. Additional evidence for the origin of the fluorescence (FL) bands of our polymers associated with triazine emission comes from the position of their maxima. Changes in the structural assembly of diimide segment of PI1–PI3 affected the FL spectra in terms of emission wavelength (small shifts) and emission intensity. Since the main polyimide chains incorporating triazine heterocycle adopt different conformations, it was expected to obtain some differences in the FL maxima. In this context, it was found that PI3 with the most flexible main chains emitted more intense light at a lower wavelength (528 nm) compared to the more rigid polymers, PI1 and PI2, that released light of lower intensity at 534 and 539 nm, respectively.

In a condensed form, the fluorescence of PI1–PI3 was almost quenched (Figure 8b), mostly due to the molecular aggregation that led to non-radiative decay by intermolecular energy transfer. This behavior is opposite to that found for related DOPO-containing polyimides [46] which displayed a strong fluorescence emission in film state, with maximum emission wavelength at about 510 nm. Thus, it can be assumed that 1,3,5-triazine brought the most significant contribution to florescence suppression of present polyimides in solid state.

### 2.5. Cyclic Voltammetry (CV) Measurements

**Figure 9:**
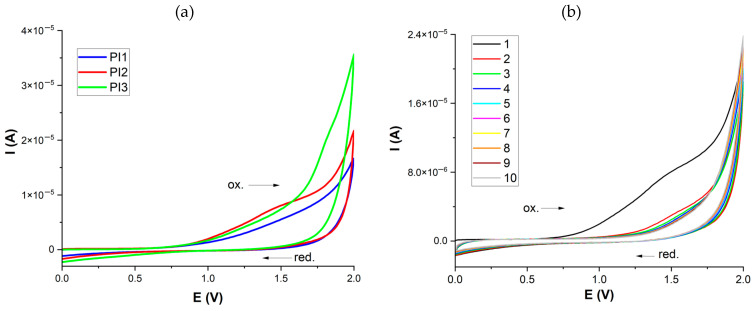
CV curves of PI1–PI3-modified ITO electrodes (**a**) and repeated CV scans of PI2-modified ITO electrode (**b**) in the anodic region.

**Figure 10:**
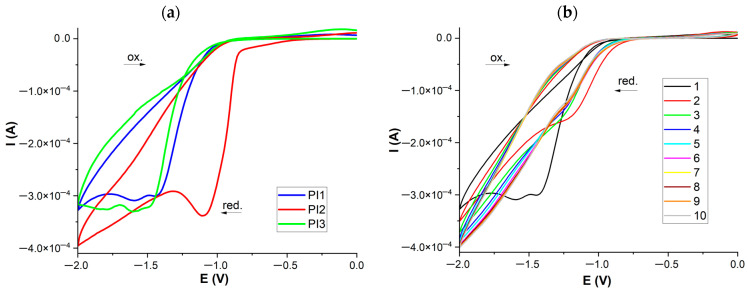
CV curves of PI1–PI3-modified ITO electrodes (**a**) and repeated CV scans of PI1-modified ITO electrode (**b**) in the cathodic region.

The investigated polymers contain strong electron-withdrawing imide, DOPO, and 1,3,5-triazine heterocycles capable of experiencing electrochemical reduction processes, hence, their electroactivity was investigated by cyclic voltammetry (CV). All CV measurements were performed on polymer-modified ITO-coated glass as the working electrode in an electrochemical cell equipped with Ag/AgCl as the reference electrode, and Pt wire as the counter electrode, in 0.1 M tetrabuthylammonium (TBAP)/acetonitrile (ACN) as the electrolyte system. The CV curves of the investigated DOPO- and triazine-containing polyimides as electrode materials at a scan rate of 50 mV/s are displayed in Figure 9 and Figure 10.

Although PI1–PI3 do not contain electron donating units, their electroactivity was screened in the anodic region to survey the existence of any possible oxidation processes. According to Figure 9a, all polyimides displayed a broad CV band centered at 1.55, 1.48, and 1.37 V vs. Ag/AgCl for PI1, PI2, and PI3, respectively, that can be associated with electron release and formation of radical cations at the NH group of residual, uncyclized amidic acid groups. Similar results were obtained on related polyimides containing triazine heterocycle having hyperbranched structure [55], when clear oxidation peaks appeared in CV curves at different potentials under positive sweeps. However, no comment was provided with respect to the origin of these peaks. The radical cations of PI1–PI3 formed during oxidation were unstable and deactivated in the next CV scans (Figure 9b), denoting weak electroactivity of these polymers in the anodic region. This was expected since the C=O unit of the amide group withdraws electrons, which are no longer available to restore the neutral molecules, and thus, the oxidation processes of the studied polymers are completely irreversible.

The expected *n*-doping-type character of these polymers is further demonstrated and discussed on the basis of the electroactivity detected in the cathodic region. Hence, all polyimides displayed electrochemical reduction processes that were observed at relatively low potentials (Figure 10a), varying between −1.1 V and −1.48 V vs. Ag/AgCl (the first reduction peak). 

Since the polymers contain three structural elements with electron accepting capability, namely phthalimide, 1,3,5-trizine, and DOPO, a strong competition between their reduction takes place. This is clearly seen in the case of PI3 where three redox peaks are registered. The first peak in the cathodic region is tentatively associated with phthalimide reduction and generation of radical anions upon acceptance of electrons. The reason why all electron-accepting units of PI2 are reduced in a single step can be found if the stability of the formed radical anions upon reduction is considered. The electron-withdrawing CF_3_ groups mostly stabilized these electrochemical species, and therefore, further reduction did not occur. The presence of more reduction peaks in the case of PI1 and PI3 provides an obvious example for the instability of the formed radical anions induced by the electron donor effect of ether and C(CH_3_)_2_ bridging groups from their diimide segments. These experienced further reductions, most likely at the C=O group of DOPO and 1,3,5-triazine. However, their reduction happened only in the first scan with no signature in the next scans, when only a broad peak appeared (Figure 10b), which mostly could be due to the reduction in newly formed entities after radical anions coupling or involvement in secondary reactions. Nevertheless, since the electrochemical activities of PI1–PI3 are dominated by the electroactivity of phthalimide moiety as a function of the surrounding substituents (DOPO and 1,3,5-triazine), these polymers can be considered valuable candidates for use as *n*-type materials in optoelectronic devices.

## 3. Materials and Methods

### 3.1. Materials

9,10-Dihydro-9-oxa-10-phosphaphenantrene-10-oxide (DOPO) and 2-vinyl-4,6-diamino-1,3,5-triazine were purchased from TCI Europe. 1,8-Diazabicyclo [5.4.0]undec-7-ene (DBU), 4,4′-oxydiphthalic anhydride (97%, ODPA), 4,4′-(hexafluoroisopropylidene)-diphthalic anhydride (99%, 6FDA), 4,4′-(4,4′-Isopropylidenediphenoxy)bis(phthalic anhydride) (97%, 6HDA), tetrabutylammonium perchlorate (≥99.0%, TBAP), and indium tin oxide coated glass slide (ITO) with surface resistivity 15–25 Ω/sq were purchased from Sigma-Aldrich. Prior polymer synthesis, ODPA, 6FDA, and 6HDA were purified by heating at 200 °C for 6 h in a heating oven. *N*-methyl pyrrolidinone (NMP, anhydrous, 99.5%), N,N’-dimethylformamide (DMF, HPLC grade), N,N’-dimethylacetamide (DMAc, HPLC grade), dimethyl sulfoxide (DMSO anhydrous, ≥99.9%), acetonitrile (ACN, anhydrous, 99.8%), toluene, and ethanol (analytical standard) were bought from different commercial sources including Roth, Fluka, TCI Europe, Sigma-Aldrich, and used without purification.

### 3.2. Synthesis

The aromatic diamine 6-(2-(4,6-diamino-1,3,5-triazin-2-yl)ethyl)dibenzo[c,e][1,2] oxaphosphinine 6-oxide (TE-DOPO) was obtained following the addition reaction of DOPO to 2-vinyl-4,6-diamino-1,3,5-triazine in toluene, by using DBU as catalyst, as reported earlier [42].

FTIR (KBr, cm^−1^): 3297, 3103 (N–H), 3062 (Ar–H), 1647 (C=C), 1536 (triazine ring), 1438 (P–CH2), 1182 (P=O), 912 (P–O–Ph).

^1^H-NMR (DMSO-d6), δ (ppm): 6.55 (s, 4H, NH_2_), 7.21–7.30 (m, 2H, Ar–H), 7.40–7.44 (t, 1H, Ar–H), 7.54–7.59 (t, 1H, Ar–H), 7.55–7.76 (t, 1H, Ar–H), 7.86–7.91 (t, 1H, Ar–H), 8.12–8.19 (m, 2H, Ar–H).

^31^P NMR (DMSO-d6), δ (ppm): 37.65.

Three aromatic polyimides denoted as PI1, PI2, and PI3 were obtained by one-step solution polycondensation reaction of diamine TE-DOPO with one of the three commercial aromatic dianhydrides: ODPA, 6FDA, or 6HDA, as shown in Scheme 1.

The reactions were carried out in NMP as solvent, and in the presence of benzoic acid as catalyst, at a concentration of 15%, according to the illustrative example further provided for PI3. First, 2 g (0.00567 mol) of TE-DOPO and 13 mL of NMP were added into a 100 mL round-bottom three-necked flask fitted with a nitrogen inlet and outlet. After that, the reaction mixture was heated to 120 °C until the dissolution of the diamine occurred, followed by the addition of 2.949 g (0.00567 mol) of 6HDA, 0.0494 g benzoic acid, and another 15 mL of NMP. The solution reaction was continuously stirred at the reflux temperature of NMP under a strong stream of nitrogen for long time period. The polyimide PI3 was obtained after precipitation in water of the resulting solution, filtration and drying at 100 °C for 6 h in a vacuum oven. The purification of the dark brown solid was performed by Soxhlet extraction with ethanol as solvent, for 48 h. Polyimides PI1 and PI2 were obtained in a similar way by replacing 6HDA with ODPA or 6FDA, respectively, in the form of dark brown powders, as shown in Scheme 1.

PI1:

FTIR (ATR, cm^−1^): 3064 (C–H aromatic), 2952, 2894 (C–H aliphatic), 1765 (C=O imide asymmetric), 1704 (C=O imide symmetric), 1608 (C=C aromatic), 1540 (triazine ring), 1475 (P–Ar), 1436 (P–CH_2_), 1378 (C–N imide), 1227 (Ar–O–Ar), 1145 (P=O), 918 (P–O–Ph), 754 (C–N ring deformation).

^31^P-NMR (DMSO-d6, ppm): 37.04, 32 (broad)

GPC (DMF, polystyrene standard): Mn = 151,700 g/mol, Mw = 354,500 g/mol, Mw/Mn =2.34.

PI2:

FTIR (ATR, cm^−1^): 3080 (C–H aromatic), 2951, 2897 (C–H aliphatic), 1777 (C=O imide asymmetric), 1701 (C=O imide symmetric), 1606 (C=C aromatic), 1541 (triazine ring), 1472 (P–Ar), 1434 (P–CH_2_), 1377 (C–N imide), 1209 (C–F), 1140 (P=O), 916 (P–O–Ph), 717 (C–N ring deformation).

^31^P-NMR (DMSO-d6, ppm): 36.98, 32.94 (broad)

GPC (DMF, polystyrene standard): Mn = 301,700 g/mol, Mw = 693,900 g/mol, Mw/Mn =2.30.

PI3:

FTIR (ATR, cm^−1^): 3060 (C–H aromatic), 2965, 2898 (C–H aliphatic), 1764 (C=O imide asymmetric), 1700 (C=O imide symmetric), 1607 (C=C aromatic), 1542 (triazine ring), 1472 (P–Ar), 1436 (P–CH_2_), 1377 (C–N imide), 1266 (Ar–O–Ar), 1146 (P=O), 920 (P–O–Ph), 748 (C–N ring deformation).

^31^P-NMR (DMSO-d6, ppm): 37.02, 33.08 (broad).

GPC (DMF, polystyrene standard): Mn = 274,200 g/mol, Mw = 599,000 g/mol, Mw/Mn = 2.19.

### 3.3. Film Preparation

The thin films were obtained from 0.5 wt.% polyimide solutions in NMP which were coated onto glass plates (for use in SEM analysis), quartz (for use in UV-Vis and fluorescence measurements), and indium tin oxide-coated glass (for CV studies) by using the drop-casting technique. The solvent removal was accomplished by subjecting the as-prepared plates to a programmed thermal treatment: 50, 100, 150, 200, and 250 °C, at each temperature for 1 h.

### 3.4. Measurements

The Fourier transform infrared spectroscopy (FTIR) measurements were performed on a FT-IR Bruker Vertex 70 spectrophotometer (Bruker, Ettlingen, Germany) in the ATR mode, by using powder samples.

The NMR spectra were measured with a Bruker Avance III 400 spectrometer (Bruker, Rheinstetten, Germany), operating at 400 MHz for ^1^H and 62 MHz for ^31^P. The chemical shifts are expressed in δ units (ppm) with respect to the residual peak of the solvent ^1^H: DMSO-d_6_ 2.512 ppm.

Gel permeation chromatography (GPC) was used to obtain the average molecular weights of the polymers by using a ParSEC Chromatography Ver. 5.67 Brookhaven Instruments Corp. (Holtsville, NY, USA) apparatus equipped with refraction and UV detectors and PL Mixed C Column. Measurements were performed with polymer solutions of 0.5% concentration in DMF as solvent. The calibration was accomplished with polystyrene standards of known molecular weights.

The UV-Vis spectra of polyimides were performed on a Shimadzu UV-1280 spectrophotometer (Kyoto, Japan), by using NMP solutions of 1 × 10^−6^ M concentrations and thin films coated onto quartz plates.

The fluorescence spectra of polyimides were recorded on a Shimadzu RF-6000 spectrofluorophotometer (Kyoto, Japan). The measurements were carried out with the same polyimide solutions and thin films used for registration of the UV-Vis absorption spectra.

The wide X-ray powder diffraction (XRD) spectra were measured on a Bruker AD8 Avance diffractometer (Billerica, MA, USA), by using Ni-filtered Cu-Kα radiation (λ = 0.1541 nm) at 36 kV and 30 mA. All diffractograms were registered at room temperature, in the range of 2–40 (2θ degrees) on polyimide powder and are reported as smoothed spectra.

The morphology of the thin polyimide films was evaluated by using a Verios G4 UC Scanning Electron Microscope (SEM) from Thermo Fisher Scientific (Waltham, MA, USA). The samples were sputtered with Pt in order to obtain a better image resolution of the film surface.

The optical microscopy photographs were taken using an Olympus BH-2 polarized light microscope (Shinjuku, Tokyo, Japan) at a magnification of 40×, at room temperature.

The differential scanning calorimetry (DSC) measurements were performed on polyimide samples in the form of powders using a Mettler Toledo DSC-type device (Mettler Toledo, Greifensee, Switzerland), by heating 3–5 mg of each sample from 25 to 300 °C, with a heating rate of 10 °C/min, under nitrogen atmosphere.

The thermogravimetric analysis (TGA) was also recorded on polyimide powders, with a Mettler Toledo TGA-SDTA851e device (Mettler Toledo, Greifensee, Switzerland), in nitrogen atmosphere. The mass of the tested samples was in the range of 2.1–3.3 mg, while the heating was applied with a rate of 10 °C/min, between 25 and 800 °C.

The cyclic voltammetry (CV) experiments were performed on a Potentiostat-Galvanostat (PG581, Uniscan Instruments, Buxton, United Kingdom) using 0.1 M TBAP/ACN as the electrolyte system. The CV curves were obtained in a classical electrochemical cell equipped with three electrodes: Pt wire, from Radiometer Analytical with ø = 1 mm and surface area of 0.785 mm^2^ as the auxiliary electrode, ITO coated with polyimide as the working electrode, and saturated Ag/AgCl as the reference electrode. All measurements were performed at a scan rate of 50 mV/s in air, at room temperature, after purging the electrolyte solution with N_2_ stream.

## 4. Conclusions

With the aim to develop new appealing materials with multifunctional properties, we synthesized and investigated high-performance polyimides containing DOPO-substituted 1,3,5-triazine bulky moiety. This structural unit, along with some flexible groups were introduced to tailor the flexibility of the polyimide chains. This molecular design led to excellent organo-solubility in polar organic solvents, which allowed polymer processing from solution into thin films with grain-like morphology. Moreover, due to the synergetic effect of imide, DOPO, and 1,3,5-triazine, these polyimides were endowed with a black appearance, exhibiting a UV-Vis absorption spectrum extended on the overall UV-Vis domain. Accordingly, the thin polymer films were almost opaque, enabling optical transmittance greater than 18% at 500 nm. Because of the presence of versatile functional groups, the polyimides showed good thermal stability, with no glass transition temperature up to 300 °C, as well as green fluorescence under excitation with UV light. The different linkages from the diimide segment had a significant role in the modulation of the electrochemical behavior of the polyimides, mainly due to the electronic effects promoted by these units. The cyclic voltammetry evidenced complex reduction behavior, affected by the stability of the formed radical anions. Nevertheless, these polymers can be considered to be among the potential candidates for use as *n*-type materials. With the attractive properties reported here, these polymers may find applications as advanced materials in electronics and (opto)electronics, as well as high-temperature materials in engineering fields.

## Figures and Tables

**Table 1:** Solubility of polyimides PI1–PI3.

Polyimide	Solvent							
	NMP	DMAc	DMSO	DMF	EtOH	CHCl_3_	DCM	Acetone
PI1	++	++	++	++	−	−	−	−
PI2	++	++	++	++	−	−	−	−
PI3	++	++	++	++	−	−	−	−

++, soluble at room temperature; −, insoluble even on heating.

**Table 2:** Molecular weight values and thermal stability parameters for PI1, PI2, and PI3.

Polyimide	Mn (g/mol)	Mw (g/mol)	Mw/Mn	T_5_ (°C)	T_max_ (°C)	W_800_ (%)
PI1	151,700	354,500	2.34	336	490	46.44
PI2	301,700	693,900	2.30	352	516/691	27.18
PI3	274,200	599,000	2.19	339	499	53.05

Mn, number average molecular weight; Mw, weight average molecular weight; T_5_, temperature of 5% weight loss; T_max_, maximum decomposition temperature; W_800_, char residue at 800 °C.

## Data Availability

The data presented in this study are available on request from the corresponding author.

## Sample Availability

Samples of the compounds may be available from the authors on request.

## References

[1] Liaw D.J., Wang K.L., Huang Y.C., Lee K.R., Lai J.Y., Ha C.S. (2012). Advanced polyimide materials: Syntheses, physical properties and applications. Prog. Polym. Sci..

[2] Ni H.J., Liu J.G., Wang Z.H., Yang S.Y. (2015). A review on colorless and optically transparent polyimide films: Chemistry, process and engineering applications. J. Ind. Eng. Chem..

[3] Ding Y., Hou H., Zhao Y., Zhu Z., Fong H. (2016). Electrospun polyimide nanofibers and their applications. Prog. Polym. Sci..

[4] Yi L., Huang W., Yan D. (2017). Polyimides with Side Groups: Synthesis and effects of side groups on their properties. J. Polym. Sci. Part A Polym. Chem..

[5] Zhuang Y., Seong J.G., Lee Y.M. (2019). Polyimides containing aliphatic/alicyclic segments in the main chains. Progr. Polym. Sci..

[6] Xu Z., Croft Z.L., Guo D., Cao K., Liu G. (2021). Recent development of polyimides: Synthesis, processing, and application in gas separation. J. Polym. Sci..

[7] Wu Z., He J., Yang H., Yang S. (2022). Progress in aromatic polyimide films for electronic applications: Preparation, structure and properties. Polymers.

[8] Yi C., Li W., Shi S., He K., Ma P., Chen M., Yang C. (2020). High-temperature-resistant and colorless polyimide: Preparations, properties, and applications. Solar Energy.

[9] Fu M.C., Higashihara T., Ueda M. (2017). Recent progress in thermally stable and photosensitive polymers. Polym. J..

[10] Damaceanu M.D., Constantin C.P., Bejan A.E., Mihaila M., Kusko M., Diaconu C., Mihalache I., Pascu R. (2019). Heteroatom-mediated performance of dye-sensitized solar cells based on T-shaped molecules. Dyes Pigm..

[11] Constantin C.P., Lisa G., Damaceanu M.D. (2021). Assessing the electrical characteristics of p-n heterojunction prototype diodes realized with n-type polyimide materials. Macromolecules.

[12] Butnaru I., Constantin C.P., Damaceanu M.D. (2023). Optimization of triphenylamine-based polyimide structure towards molecular sensors for selective detection of heavy/transition metal ions. J. Photochem. Photobiol. A-Chem..

[13] Yuan H., Yang S., Liu X., Wang Z., Ma L., Hou K., Yang Z., Wang J. (2017). Polyimide-based lubricating coatings synergistically enhanced by MoS2@HCNF hybrid. Compos. Part A Appl. Sci. Manuf..

[14] Liu Y., Ge X., Li J. (2020). Graphene lubrication. Appl. Mater. Today.

[15] Bruma M., Damaceanu M.D., Muller P. (2009). Comparative study of polyimides containing oxadiazole and ether groups. High Perform. Polym..

[16] Butnaru I., Varganici C.-D., Pinteala M., Lehner S., Bruma M., Gaan S. (2018). Thermal decomposition of polyimides containing phosphine-oxide units. J. Anal. Appl. Pyrolys..

[17] Butnaru I., Sava I., Damaceanu M.-D. (2020). Exploring the impact of triphenylmethane incorporation on physical properties of polyimides with emphasis on optical and halochromic behaviour. Polymer.

[18] Butnaru I., Chiriac A.P., Asandulesa M., Sava I., Lisa G., Damaceanu M.D. (2021). Tailoring poly(ether-imide) films features towards high performance flexible substrates. J. Ind. Eng. Chem..

[19] Damaceanu M.-D., Constantin C.-P., Bruma M., Begunov R.S. (2018). The photo-optical and electrochemical activity promoted by trifluoromethyl-substituted and ortho-catenated triphenylamine core in poly(ether-imide)s. Polymer.

[20] Guerrini M., Delgado Aznar E., Cocchi C. (2020). Electronic and optical properties of protonated triazine derivatives. J. Phys. Chem. C.

[21] Prokhorov A.M., Kozhevnikov D.N., Gribble G.W., Joule J.A. (2012). Chapter 6.3—Triazines, Tetrazines, and Fused Ring Polyaza Systems, in Progress in Heterocyclic Chemistry.

[22] Omer K.M., Ku S., Chen Y., Wong K., Bard A.J. (2010). Electrochemical behavior and electrogenerated chemiluminescence of star-shaped D−A compounds with a 1,3,5-triazine core and substituted fluorene arms. J. Am. Chem. Soc..

[23] Zhong H., Lai H., Fang Q. (2011). New conjugated triazine based molecular materials for application in optoelectronic devices: Design, synthesis, and properties. J. Phys. Chem. C.

[24] Zhang Z., Liu R., Zhu X., Li Y., Chang J., Zhu H., Ma L., Lv W., Guo J. (2014). Synthesis and luminescent properties of star-burst D-π-A compounds based on 1,3,5 triazine core and carbazole end-capped phenylene ethynylene arms. J. Lumin..

[25] Ziarani G.M., Moradi R., Lashgari N., Kruger H.G. (2018). Metal-Free Synthetic Organic Dyes.

[26] Li Z.T., Zhou J.Y., Xu R.F., Liu S.P., Wang Y.K., Li P., Wu W.T., Wu M.B. (2016). Synthesis of three dimensional extended conjugated polyimide and application as sodium-ion battery anode. Chem. Eng. J..

[27] Mokhtari N., Dinari M., Rahmanian O. (2019). Novel porous organic triazine-based polyimide with high nitrogen levels for highly efficient removal of Ni(II) from aqueous solution. Polym. Int..

[28] Bugel S., Hoang Q.D., Spiess A., Sun Y.Y., Xing S.H., Janiak C. (2021). Biphenyl-based covalent triazine framework/Matrimid(R) mixed-matrix membranes for CO_2_/CH_4_ separation. Membranes.

[29] Zhao G.F., Li H.N., Gao Z.H., Xu L.F., Mei Z.Y., Cai S., Liu T.T., Yang X.F., Guo H., Sun X.L. (2021). Dual-active-center of polyimide and triazine modified atomic-layer covalent organic frameworks for high-performance Li storage. Adv. Funct. Mater..

[30] Dai H.C., Zou J.C., Gao Y.B., Li Z.Y., Zhang C.Y., Zhang G.Q., Wang X.B., Wang C.L. (2022). A novel conjugated porous polymer based on triazine and imide as cathodes for sodium storage. J. Polym. Sci..

[31] Chang C.W., Lin C.H., Cheng P.W., Hwang H.J., Dai S.A. (2009). Facile and efficient preparation of phosphinate-functionalized aromatic diamines and their high-Tg polyimides. J. Polym. Sci. Part. A Polym. Chem..

[32] Jian S.J., Ding C.H., Yang T., Zhang C.W., Hou H.Q. (2018). Effect of trace diphenyl phosphate on mechanical and thermal performance of polyimide composite films. Compos. Com..

[33] Wang D., Yu J.J., Duan G.G., Liu K.M., Hou H. (2020). Electrospun polyimide nonwovens with enhanced mechanical and thermal properties by addition of trace plasticizer. J. Mater. Sci..

[34] Wu L., Wu X., Qi H.R., An Y.C., Jia Y.J., Zhang Y., Zhi X.X., Liu J.G. (2021). Colorless and transparent semi-alicyclic polyimide films with intrinsic flame retardancy based on alicyclic dianhydrides and aromatic phosphorous-containing diamine: Preparation and properties. Polym. Adv. Technol..

[35] Hamciuc C., Hamciuc E., Serbezeanu D., Vlad-Bubulac T., Cazacu M. (2011). Phosphorus-containing poly(ester-imide)-polydimethylsiloxane copolymers. Polym. Int..

[36] Agrawal S., Narula A.K. (2013). Facile synthesis of new thermally stable and organo-soluble polyamide-imides from phosphorus-containing aromatic amines and various dianhydrides. J. Polym. Eng..

[37] Cakir M., Karatas S., Menceloglu Y., Kayaman-Apohan N., Gungor A. (2008). Phosphorus-containing sulfonated polyimides for proton exchange membranes. Macromol. Chem. Phys..

[38] Ghorai A., Banerjee S. (2023). Phosphorus-containing aromatic polymers: Synthesis, structure, properties and membrane-based applications. Progr. Polym. Sci..

[39] Song G.H., Li X.S., Jiang Q.Y., Mu J.X., Jiang Z.H. (2015). A novel structural polyimide material with synergistic phosphorus and POSS for atomic oxygen resistance. RSC Adv..

[40] Wu B.H., Zhang Y., Yang D.Y., Yang Y.B., Yu Q., Che L., Liu J.G. (2019). Self-healing anti-atomic-oxygen phosphorus-containing polyimide film via molecular level incorporation of nanocage trisilanolphenyl POSS: Preparation and characterization. Polymers.

[41] Guan Y., Sun Y., Shang D.J. (2022). Amine-functionalized POSS cross-linked the poly(imide siloxane) block copolymers nanocomposites: Preparation, thermal properties, against atomic oxygen erosion. J. Macromol. Sci. Part A Pure Appl. Chem..

[42] Butnaru I., Fernandez-Ronco M., Czech-Polak J., Heneczkowski M., Bruma M., Gaan S. (2015). Effect of meltable triazine-DOPO additive on rheological, mechanical, and flammability properties of PA6. Polymers.

[43] Chatterjee R., Bisoi S., Kumar A.G., Padmanabhan V., Banerjee S. (2018). Polyimides containing phosphaphenanthrene skeleton: Gas-transport properties and molecular dynamics simulations. ACS Omega.

[44] Lin C.H., Chang S.L., Cheng P.W. (2011). Dietheramine from an alkaline-stable phosphinated bisphenol for soluble polyetherimides. Polymer.

[45] Liu Y.-L., Hsu C.-Y., Wu C.-S. (2003). Polyimides possessing bulky phosphorus groups: Synthesis and characterization. J. Appl. Polym. Sci..

[46] Homocianu M., Serbezeanu D., Lisa G., Brebu M., Vlad-Bubulac T. (2022). Optical and flame-retardant properties of a series of polyimides containing side chained bulky phosphaphenanthrene units. Int. J. Mol. Sci..

[47] Zhai Y., Yang Q., Zhu R., Gu Y. (2007). The study on imidization degree of polyamic acid in solution and ordering degree of its polyimide film. J. Mater. Sci..

[48] Uchida S., Ishige R., Takeichi T., Ando S. (2017). Promotion of thermal imidization of semi-aliphatic polyimide precursors by incorporation of polyethylene glycol and their modified solid structures. J. Photopolym. Sci. Technol..

[49] Rahnamoun A., Engelhart D.P., Humagain S., Koerner H., Plis E., Kennedy W.J., Cooper R., Greenbaum S.G., Hoffmann R., van Duin A.C.T. (2019). Chemical dynamics characteristics of Kapton polyimide damaged by electron beam irradiation. Polymer.

[50] Wakita J., Jin S., Shin T.J., Ree M., Ando S. (2010). Analysis of molecular aggregation structures of fully aromatic and semialiphatic polyimide films with synchrotron grazing incidence wide-angle X-ray scattering. Macromolecules.

[51] Hajibeygi M., Habibnejad N., Shabanian M., Khonakdar H.A. (2021). Fabrication and study of thermal and combustion resistance of DOPO-functionalized polyamide reinforced with organo-modified Mg(OH)_2_ nanoparticles. Polym. Int..

[52] Damaceanu M.-D., Bacosca I., Bruma M., Robison J., Rusanov A. (2009). Heterocyclic polyimides containing siloxane groups in the main chain. Polym. Int..

[53] Zhan Y., Guan X., Ren E., Lin S., Lan J. (2019). Fabrication of zeolitic imidazolate framework-8 functional polyacrylonitrile nanofibrous mats for dye removal. J. Polym. Res..

[54] Sun D., Si C., Wang T., Zysman-Colman E. (2022). 1,3,5-Triazine-functionalized thermally activated delayed fluorescence emitters for organic light-emitting diodes. Adv. Photonics Res..

[55] Li Z., Kou K., Zhang J., Zhang Y., Wang Y., Pan C. (2017). Solubility, electrochemical behavior and thermal stability of polyimides synthesized from 1,3,5-triazine-based diamine. J. Mater. Sci. Mater. Electron..

